# Can we increase efficiency of CT lung cancer screening by combining with CVD and COPD screening? Results of an early economic evaluation

**DOI:** 10.1007/s00330-021-08422-7

**Published:** 2022-01-01

**Authors:** Carina M. Behr, Hendrik Koffijberg, Koen Degeling, Rozemarijn Vliegenthart, Maarten J. IJzerman

**Affiliations:** 1 Health Technology and Services Research, Faculty of Behavioural and Management Science, University of Twente, Drienerlolaan 5, 7522 NB Enschede, The Netherlands; 2 Cancer Health Services Research, University of Melbourne Centre for Cancer Research, Faculty of Medicine, Dentistry and Health Sciences, The University of Melbourne, Parkville, Melbourne VIC 3010 Australia; 3 Cancer Health Services Research, Centre for Health Policy, Melbourne School of Population and Global Health, Faculty of Medicine, Dentistry and Health Sciences, The University of Melbourne, Parkville, Melbourne VIC 3010 Australia; 4 Dept of Radiology, University of Groningen, University Medical Centre Groningen, Hanzeplein 1, 9713 GZ Groningen, The Netherlands

**Keywords:** Cost–benefit analysis, Mass screening, Lung neoplasms, Pulmonary disease, chronic obstructive, Cardiovascular diseases

## Abstract

**Objectives:**

Estimating the maximum acceptable cost (MAC) per screened individual for low-dose computed tomography (LDCT) lung cancer (LC) screening, and determining the effect of additionally screening for chronic obstructive pulmonary disease (COPD), cardiovascular disease (CVD), or both on the MAC.

**Methods:**

A model-based early health technology assessment (HTA) was conducted to estimate whether a new intervention could be cost-effective by calculating the MAC at a willingness-to-pay (WTP) of €20k/quality-adjusted life-year (QALY) and €80k/QALY, for a population of current and former smokers, aged 50–75 years in The Netherlands. The MAC was estimated based on incremental QALYs gained from a stage shift assuming screened individuals are detected in earlier disease stages. Data were obtained from literature and publicly available statistics and validated with experts.

**Results:**

The MAC per individual for implementing LC screening at a WTP of €20k/QALY was €113. If COPD, CVD, or both were included in screening, the MAC increased to €230, €895, or €971 respectively. Scenario analyses assessed whether screening-specific disease high-risk populations would improve cost-effectiveness, showing that high-risk CVD populations were more likely to improve economic viability compared to COPD.

**Conclusions:**

The economic viability of combined screening is substantially larger than for LC screening alone, primarily due to benefits from CVD screening, and is dependent on the target screening population, which is key to optimise the screening program. The total cost of breast and cervical cancer screening is lower (€420) than the MAC of Big-3, indicating that Big-3 screening may be acceptable from a health economic perspective.

**Key Points:**

*• Once-off combined low-dose CT screening for lung cancer, COPD, and CVD in individuals aged 50–75 years is potentially cost-effective if screening would cost less than €971 per screened individual.*

*• Multi-disease screening requires detailed insight into the co-occurrence of these diseases to identify the optimal target screening population.*

*• With the same target screening population and WTP, lung cancer-only screening should cost less than €113 per screened individual to be cost-effective.*

**Supplementary Information:**

The online version contains supplementary material available at 10.1007/s00330-021-08422-7.

## Introduction

In The Netherlands, lung cancer (LC) accounts for over 13,000 diagnoses and 10,000 deaths annually [1]. Given its high disease burden, there is interest in early detection through population-based screening using low-dose computed tomography (LDCT) to reduce LC-related mortality.

Several studies such as the largest National Lung Screening Trial (NLST) and Dutch-Belgian Randomized Lung Cancer Screening (NELSON) trial demonstrated the clinical benefits of LC screening for an at-risk population [2, 3]. Additionally, recent cost-effectiveness studies were published for different countries, including the UK [4], the USA [5], Germany [6], and Canada [7]. Although the cost-effectiveness of LC screening was generally acceptable in populations aged 50–80 years with different smoking histories, the cost-effectiveness varied from €21k to 85k per life-year gained (LYG) and from €30k to 140k per quality-adjusted life-year (QALY), which provides evidence that screening is cost-effective in some subgroups relative to given willingness-to-pay (WTP) thresholds [4–8].

The published cost-effectiveness studies focused on LC screening only. However, screening for additional diseases simultaneously could be economically attractive, particularly for diseases with an indolent start and shared risk factors (9). Chest LDCT, used in LC screening, can simultaneously detect early stages of chronic obstructive pulmonary disease (COPD) through emphysema or air trapping evaluation and high cardiovascular disease (CVD) risk based on coronary calcium scoring; both diseases pose a large burden on Western societies [9]. LC, COPD, and CVD together are also called the Big-3 [9].

Although the value of LDCT screening of COPD and CVD is still under debate [10, 11], the additional screening for these diseases within a lung cancer screening program could further improve the health outcomes of lung cancer screening at marginal additional costs as there is evidence indicating that many individuals in lung cancer screening programs have high, unrecognised CVD risks [12]. COPD and CVD are both diseases that can be detected and acted upon in the early stages. There are as yet no clinical trials with outcome results that prove the effectiveness of COPD or CVD screening with LDCT as a source of evidence. Therefore, this study is conducted as an early health technology assessment using the limited evidence available. Using this limited evidence in modelling can be beneficial to estimate if combination screening could offer an attractive alternative to screening for LC only. Health economists proposed methods using expected health benefits of combination screening, additional cost, and a certain willingness-to-pay (WTP) threshold, to estimate the maximum acceptable cost (MAC) under optimistic circumstances. If the anticipated screening cost is higher than the MAC, the program is unlikely to be cost-effective. Such analyses, called headroom analyses, have been proven useful to inform decisions on further research [13–16] and are preferred during the intervention and evidence development, to optimize further data collection and to more accurately estimate the long-term health economic impact when more clinical evidence becomes available.

This study aims to estimate the MAC per screened individual in The Netherlands for LC screening and to determine the effect of additional screening for COPD, CVD, or both.

## Materials and methods

This study compared once-off LDCT screening for LC, with the addition of CVD, COPD, or both to no-screening in a stochastic data-based analysis without the involvement of participants. Although annual and biennial screening is more common, a single screening round is considered a starting point and in some cases could be more cost-effective than repeated screening [17].

The optimistic MAC for screening was calculated for two WTP thresholds based on estimated health benefits and treatment costs per disease stage. These calculations were based on population-level data for disease stage-specific health outcomes and costs. The high-risk target screening population was current and former smokers aged 50–75 in The Netherlands, corresponding to the population of the NELSON trial [18]. Details on the input values with its sources, how each disease was modelled, and the scenarios are given in the supplements.

### General approach

This analysis used a stage-shift model that is relevant to progressive diseases, where screened individuals are detected in earlier disease stages than non-screened individuals [19–24]. Detection in an earlier stage increases the therapeutic window and thus health benefits. LC was classified by tumour, node, metastasis (TNM) staging [25], and COPD by the Global Initiative for Obstructive Lung Disease (GOLD) criteria [26]. Individuals at risk of CVD were grouped into three risk categories, based on risk factors, for determining the proportion of individuals per risk category experiencing CVD events [20]. The modelling of health effects after screening differs per disease. For LC, the most evidence exists and it can be assumed that screening results in a stage shift, where utility and costs of lower disease stages are assigned to more patients and later disease stages to fewer patients. Given the lack of evidence for the efficacy of COPD and CVD screening, the health effects are modelled as follows. For COPD, the assumed health effect is that a stage-specific proportion (0.2–0.3) [27] of diagnosed patients will stop smoking which slows progression (modelled by annual rate of decline in FEV1). The rates used here are comparable with smoking cessation found in the NELSON trial [28]. COPD patients who do not stop smoking are assumed not to have health benefits, but only COPD-related maintenance costs. Individuals at risk of CVD in the model may experience no CVD-event, experience a CVD-event with the related disutility and costs, or experience a fatal CVD-event with related costs. Due to preventative treatment of at-risk individuals, the probability that a fatal or non-fatal CVD event is experienced declines.

### Calculating MAC

The MAC represents the maximum cost of LDCT and organisational costs for screening to be cost-effective. The MAC or headroom [29] was calculated according to the following formula for various WTP thresholds, where direct healthcare costs for disease management were included in the *IncrementalCosts* and QALYs were included in the *EffectivenessGap* for screening compared to no-screening:

$$MAC\;for\;screening\:=\:(EffectivenessGap\;\ast\;WTP)\:-\:IncrementalCost$$

### Analysis and scenarios

The analysis was conducted in R version 3.6.1 [30]. The MAC per screened individual was calculated for the base case with two additional scenarios to assess the impact of assumptions. Depending on the diseases screened for and the screening population considered, the utility, survival, incidence rate, and costs were adjusted. Details for the scenarios are presented in Supplement Table 3, and a high-level overview is provided in this paper.

The base-case analysis estimates the MAC based on the difference between the current stage distribution in The Netherlands with no screening (A) and a realistic stage distribution (B) after screening based on literature.

### Input parameters

**Fig. 1:**
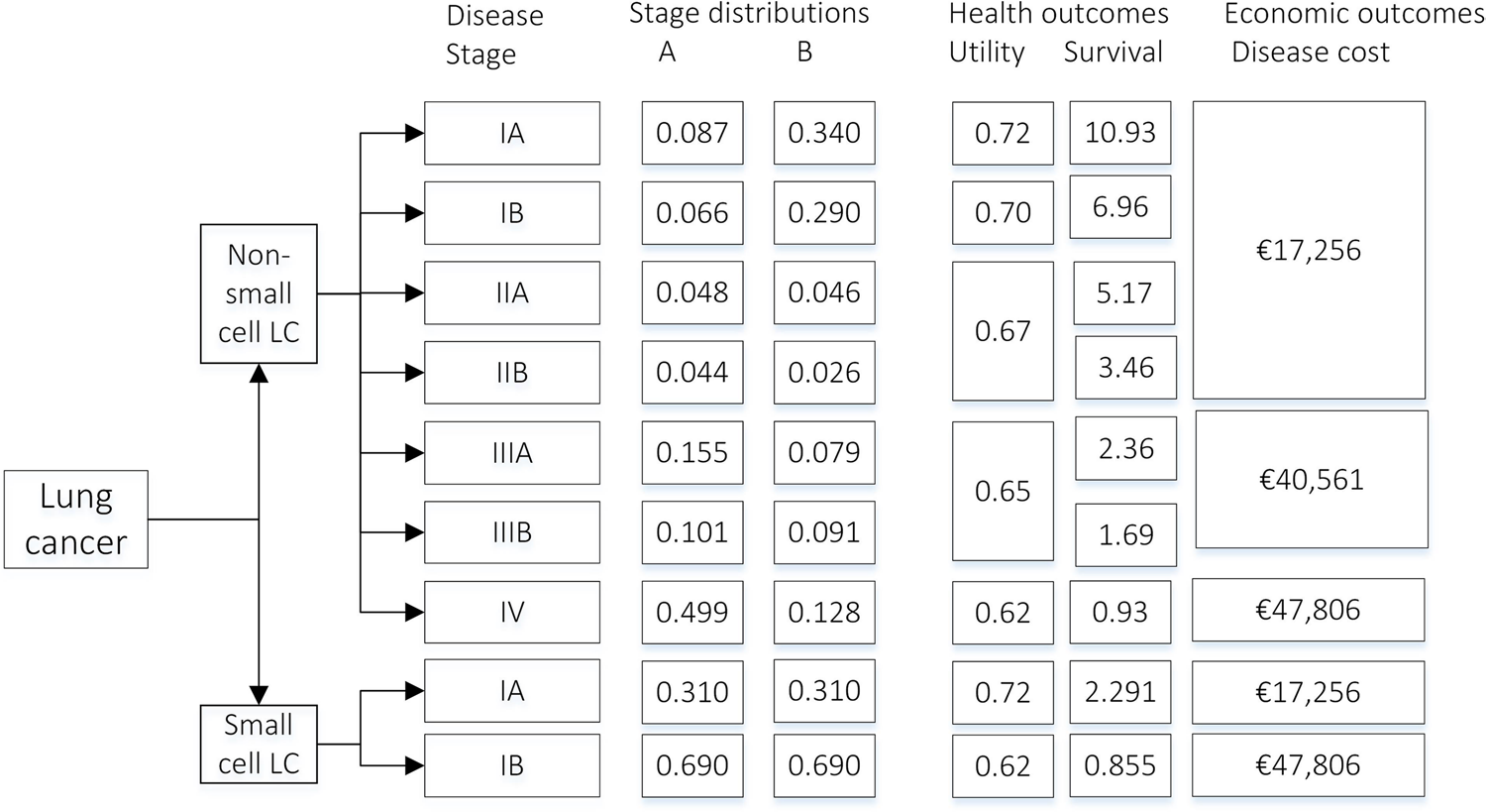
Stage distributions of LC as currently observed (A), and assuming a plausible stage distribution (B) and a stage distribution with best possible screening outcomes (C), as well as the health and economic outcomes per disease stage. Supplement-Fig. 1 presents this information for COPD and CVD as well. LC, lung cancer; COPD, chronic obstructive pulmonary disease; CVD, cardiovascular disease

Inputs for the MAC calculation included incidence rates, stage distributions of patients with and without screening, and disease-stage-specific estimates of quality of life (utility), survival, and disease management costs. The disease management cost per patient over average life expectancy for each LC or COPD stage or per CVD event included direct healthcare costs, such as cost of treatment, GP and specialist visits, and hospitalisation [31–34]. The cost of implementing screening, such as invitations and data management, was not included. Figure 1 displays the decision model with the input values used for non-small cell (NSC) LC.

For purpose of illustration, Table 1 demonstrates how the MAC can be calculated for screening for NSCLC only. Similar calculations were performed for SCLC, COPD, and CVD. The incidence rate, utility, survival, and stage distribution (with or without screening) were used to calculate the *EffectivenessGap.* These same inputs and the stage-specific disease management costs were used to calculate the *IncrementalCost*.

**Table 1:** Example of MAC calculation for non-small cell lung cancer (NSCLC) screening

	Model input: stage distributions reflecting the effect of screening		Model input: expected quality of life (measured in QALYs) and cost from diagnosis		Effect of screening	Output: MAC of screening
Non-small cell LC stage	No-screening	Screening	QALY gains compared to healthy individuals	Incremental cost of disease screening vs no-screening	QALY gains from screening ( EffectivenessGap)	WTP* × effectiveness gap − incremental costs
IA	0.087	0.340	1.080	€4,365	(0.340–0.087) × 1.080 = 0.27	0.27 × 20,000 − 4,365 = − €1,099
IB	0.055	0.290	− 1.680	€3,865	− 0.38	− €11,392
IIA	0.048	0.046	− 2.814	− €35	0.01	€147
IIB	0.044	0.025	− 3.953	− €311	0.07	€1,734
IIIA	0.155	0.079	− 4.550	− €3,083	0.35	€9,999
IIIB	0.101	0.091	− 5.005	− €406	0.05	€1,407
IV	0.499	0.128	− 5.270	− €17,736	1.96	€56,839
At the population level					∑ QALY = 0.789	∑MAC = €59,833 per LC patient
MAC per screened individual (proportion of screened individuals with NSCLC = 0.278%)					∑MAC / *N* = €166, which is the maximum acceptable cost of the screening per screened individual	

*In this example, a WTP of €20 k/QALY is used. In the analysis a WTP of €80 k/QALY is also considered, because these two thresholds are the lowest and highest thresholds used in The Netherlands, depending on disease severity (29)

*MAC* maximum acceptable cost, *NSCLC* non-small cell lung cancer, *LC* lung cancer, *QALY* quality-adjusted life-years, *WTP* willingness-to-pay

### Multiple diseases

The calculation illustrated in Table 1 for NSCLC only was extended for all considered diseases using incidence rates and by accounting for the probability of having two or three diseases simultaneously (co-occurrences). Patients with one or more Big-3 co-occurrences (e.g., NSCLC and COPD) were assumed to have the lowest QALYs (utility x survival) and the sum of the costs of the diseases that co-occur.

### Alternative scenario 1: different target populations

The MAC may change substantially when screening is implemented in different risk groups determined by age and smoking history. More detailed pieces of evidence of diagnosis within current and former smokers for specific ages are not available; therefore, the MAC was also calculated for two easily identifiable alternative high-risk groups of the Big-3 to indicate what the effect on cost-effectiveness could be and within what range the MAC could be. Firstly, current smokers aged 50–75 and secondly, all individuals in The Netherlands over 60 years old.

### Alternative scenario 2: incidence rate ranges

The base-case calculations were made using a population at risk of LC. This is logical when considering the expansion of LC screening, but might not be the most cost-effective approach for combination screening. CVD and COPD have risk factors similar to those of LC and thus, a population at increased LC risk will also have increased CVD and COPD risk. However, focusing first on CVD (or COPD) risk rather than LC risk would likely yield a target population with even higher CVD (or COPD) risk, but with much lower LC risk. The impact of such selection was illustrated in a scenario analysis by calculating the MAC for multiple combinations of Big-3 incidence rates. The following *maximum* incidence rates were chosen based on the highest reported incidence rates in subgroups found in literature: 5% for LC, based on 2.6% reported from the NELSON trial [35]; 40% for CVD, based on 31.6% of individuals older than 40 in urban areas with a high risk of CVD [36]; and 25% for COPD, based on 23% COPD incidence found in individuals over 40 years of age [37].

## Results

### Base-case

Table 2 presents the results for a screening population of current and former smokers between 50 and 75 years of age in The Netherlands, corresponding to approximately 3.5 million individuals [38, 39]. Screening for all Big-3 diseases simultaneously had the largest MAC (€971 to €3,844) per screened individual, depending on the WTP threshold. The MAC for Big-3 screening was substantially larger than screening for LC only which was €113 to €341, depending on the WTP. Note that the incremental disease management costs (or savings) are reported per screened individual, while these costs were only incurred for patients with a disease. A negative value indicates an overall cost-saving and a positive value, costs incurred. These values were driven by the cost per disease stage, which was not necessarily lower in an earlier disease stage; for example, the most expensive stage of NSCLC was stage II (Fig. 1).

**Table 2:** Headroom analysis outcomes for a screening population of current and former smokers between 50 and 75 years old

				Incremental MAC (€ per screened individual)	
		Incremental disease management costs (€ per screened individual)	Effectiveness gap (incremental QALY per screened individual)	WTP: €20 k/QALY	WTP: €80 k/QALY
Diseases screened*	Patients with disease				
LC + CVD + COPD	155,966	− 14	0.048	971	3,844
LC + CVD	136,752	− 12	0.044	895	3,546
LC + COPD	43,666	− 37	0.009	230	809
LC	13,262	− 37	0.004	113	341

^*^The + in the screening strategy refers to the diseases separately and as co-occurrence. Thus, LC + COPD refers to detecting patients with LC, or COPD, or LC and COPD

Note that the results may not appear to be exact, due to the rounding of the presented values

*MAC* maximum acceptable cost, *LC* lung cancer, *CVD* cardiovascular disease, *COPD* chronic obstructive pulmonary disease, *QALY* quality-adjusted life-years, *WTP* willingness-to-pay

These results show that screening for LC and CVD has a larger MAC than LC and COPD screening (€895 compared to €230 with a low WTP). The MAC of combined screening was not merely the sum of the MAC of screening for the three diseases separately because there is an overlap of patients with co-occurrences in each group of patients with the disease.

### Scenario analysis: impact of changing the target population

Table 3 shows the impact of targeting screening at current smokers aged 50 to 75 in The Netherlands (approximately 1.2 million individuals). In this population, the MAC is lower than in the base case for Big-3 screening for both WTP (e.g. €767 vs €971 for a WTP of €20 k/QALY). For LC-only screening, the MAC is higher in a smoking population than in the base case (€340 vs €113). The smoking population included a smaller number of patients and a proportion of patients with at least one disease than the base-case population while the relative disease incidence rates did not change substantially compared to the base-case population. This means that the incremental health benefits only applied to a small subgroup and therefore the MAC per screened individual was smaller.

**Table 3:** Headroom analysis outcomes for the smoking population of The Netherlands

				Incremental MAC (€ per screened individual)	
		Incremental disease management costs (€ per screened individual)	Effectiveness gap (incremental QALY per screened individual)	WTP: €20 k/QALY	WTP: €80 k/QALY
Diseases screened*	Patients with disease				
LC + CVD + COPD	42,662	− 88	0.034	767	2,806
LC + CVD	35,001	− 87	0.030	690	2,499
LC + COPD	25,630	− 105	0.018	466	1,546
LC	12,655	− 110	0.012	340	1,031

^*^The + in the screening strategy refers to the diseases separately and as co-occurrence. Thus, LC + COPD refers to detecting patients with LC, or COPD, or LC and COPD

Note that the results may not appear to be exact, due to the rounding of the presented values

*MAC* maximum acceptable cost, *LC* lung cancer, *CVD* cardiovascular disease, *COPD* chronic obstructive pulmonary disease, *QALY* quality-adjusted life-years, *WTP* willingness-to-pay

Table 4 shows the impact of targeting screening at a population of all individuals over the age of 60 (4.5 million individuals). In this older population, the larger number of patients and proportion of patients with a disease (220,366) compared to the base case (155,966) resulted in a larger MAC (€1,082 vs €971 for a WTP of €20 k/QALY).

**Table 4:** Headroom analysis for individuals over 60 years of age in The Netherlands

				Incremental MAC (€ per screened individual)	
		Incremental disease management costs (€ per screened individual)	Effectiveness gap (incremental QALY per screened individual)	WTP: €20 k/QALY	WTP: €80 k/QALY
Diseases screened*	Patients with disease				
LC + CVD + COPD	220,366	23	0.055	1,082	4,399
LC + CVD	201,796	24	0.052	1,028	4,185
LC + COPD	37,316	− 17	0.006	138	502
LC	8,822	− 19	0.002	58	175

^*^The + in the screening strategy refers to the diseases separately and as co-occurrence. Thus, LC + COPD refers to detecting patients with LC, or COPD, or LC and COPD

Note that the results may not appear to be exact, due to the rounding of the presented values

*MAC* maximum acceptable cost, *LC* lung cancer, *CVD* cardiovascular disease, *COPD* chronic obstructive pulmonary disease, *QALY* quality-adjusted life-years, *WTP* willingness-to-pay

The results in Table 3 and Table 4 suggest that, when screening for the Big-3 in a population of current smokers, or individuals over 60 years of age, the latter resulted in higher health benefits (0.055 vs 0.034) and is, therefore, more likely to result in a cost-effective screening program.

### Scenario analysis: impact of changes in incidence rates

The MAC was calculated for a range of LC incidence rates, in combination with a range of COPD incidence rates with CVD set to a maximum expected incidence rate, and separately, a range of CVD incidence rates with COPD set at a maximum expected incidence rate.

**Fig. 2:**
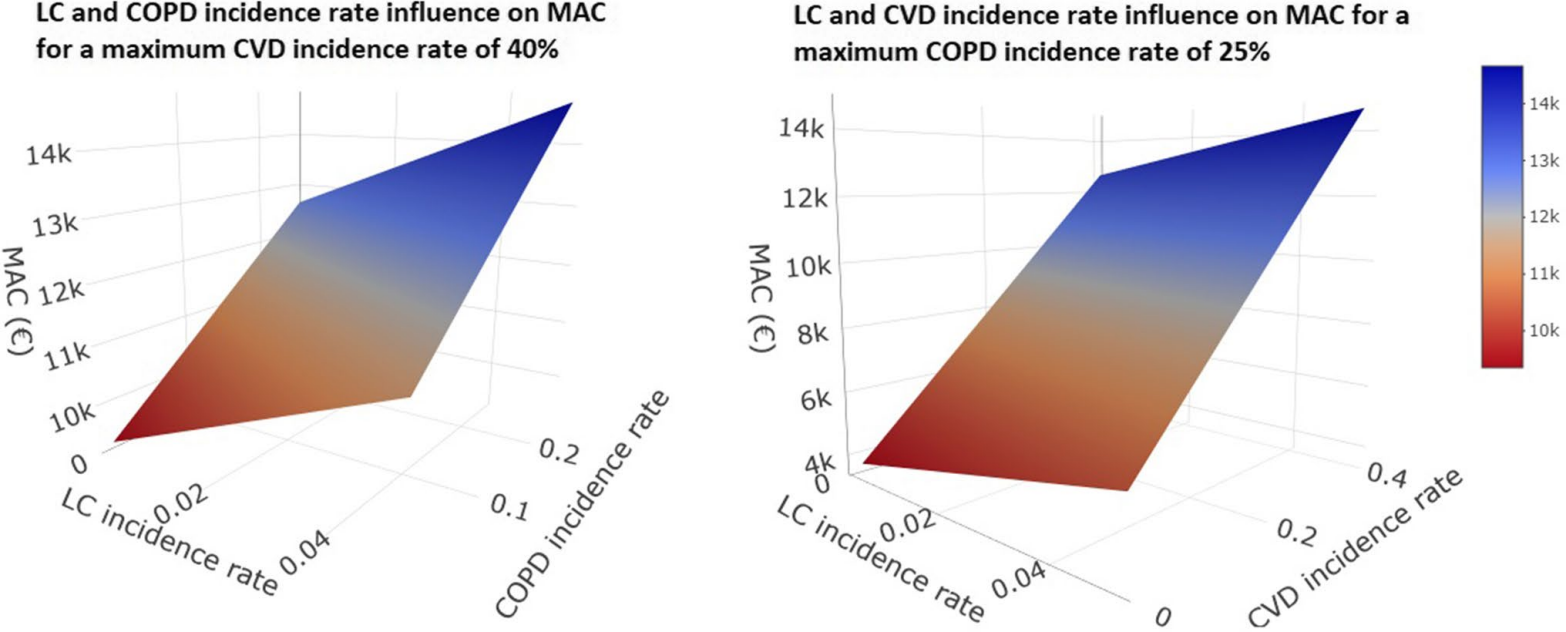
The influence of COPD and CVD incidence rate on MAC. COPD, chronic obstructive pulmonary disease; CVD, cardiovascular disease; MAC, maximum acceptable cost

The model was used to estimate the MAC for all combinations of incidence rates for two diseases at a time and indicates that the MAC increases with increasing incidence rates (Fig. 2). The MAC increased as incidences increased, with the highest MAC achieved when the incidences for all three diseases were at their maximum plausible values (top right corner of both figures), indicating the maximal benefit of screening for a population with a high incidence rate for all three diseases, with increasing likelihood of the screening being cost-effective.

## Discussion

To our knowledge, this study is the first to apply a headroom analysis estimating the MAC for a screening program. The MAC refers to an (optimistic) estimate of the upper limit of the acceptable cost per screened individual.

For Big-3 combination screening to be potentially cost-effective for a screening population of current and former smokers between ages 50 and 75, costs should be substantially less than €971 for a WTP of €20k/QALY and €3,844 for a WTP of €80k/QALY. For breast and cervical cancer screening, costs of €420 per screened individual have been estimated, after converting the currency and expressing costs in 2020 Euros based on the Dutch Consumer price index [40]. These screening costs include screening and diagnostic services, patient support, case management, program management, data management, and other smaller costs. This reference cost of breast and cervical cancer screening puts the MAC of Big-3 screening into perspective and in a positive light.

If we can assume that Big-3 screening will incur costs comparable to those of breast and cervical cancer screening. Then, the estimated MAC of €113 for LC-only screening compared to no-screening with a WTP of €20k/QALY seems low compared to previously published studies that found LC screening to be cost-effective [4–8]. However, higher WTP thresholds ranging between 21–85k€/LYG and 30–140k€/QALY were applied [4–8], which is comparable to the MAC of €335 per screened individual when evaluating LC screening at €80k/QALY.

The results showed a higher MAC for screening for LC and CVD compared to LC and COPD, which can be ascribed to the limited benefits of early detection of COPD, where the most widely used treatment and only treatment in this model was smoking cessation, which is associated with poor adherence and which delays rather than avoids disease progression.

The MAC of Big-3 screening could be enlarged if a target screening population is identified with high disease incidences and, therefore, higher average health benefits per screened individual. In this study, the trade-off between a screening population with a high risk for one disease and a population with a relatively high disease risk for all of the Big-3 was illustrated. A focus on disease risk and subsequently, higher incidence rates, improves the cost-effectiveness of this combination screening program.

Further research is required to investigate the cost-effectiveness of Big-3 screening based on prospective studies. These studies can, for instance, investigate an ideal target screening population and ideal recurrence of screening in a more in-depth analysis when evidence based on individual patient data for all three these diseases diagnosed with LDCT become available. To estimate the cost-effectiveness of Big-3 screening with more certainty, a comprehensive patient-level simulation model, populated with real-world data, would be required. In particular data such as participation rates, quality of life, and treatment outcomes of patients with co-occurrences are of importance. The adherence of individuals to screening within these target groups with high disease incidence rates might also be different when screening for different combinations of diseases.

This study had some limitations. First, the analysis assumed 100% sensitivity, specificity, and participation rate which is an unrealistic assumption but in the setting of this analysis provides an optimistic estimation which can be followed with a full cost-effectiveness analysis using real-world trial data. Second, for the base-case, all incidences in The Netherlands were assumed to occur within the defined screening population and detected in a single screening round of current and former smokers between 50 and 75 years of age which is intended to serve as a starting point for the evaluation of screening. Third, patients with co-occurrences are assumed to have the QALY (utility × survival) of the most severe disease. Fourth, incidence rates are used within the model, implying that a disease is only detected through screening within the first year of getting the disease; thereafter, the disease is always and automatically detected through current diagnostic processes. Fifth, the combination of three diseases into one screening program posed the challenge of identifying and synthesizing evidence into homogenous inputs and a simple model structure. It was challenging in this combination of diseases, where the nature of progression and curing of the Big-3 differ. Lastly, in this analysis, the impact of CVD events was based only on patients experiencing a myocardial infarction. Some of the assumptions are deliberately optimistic, which aligns with the goal of a headroom analysis, being an early-stage estimation of potential cost-effectiveness performed to filter out interventions that are certainly not cost-effective.

In conclusion, this study indicates that LDCT screening for LC, COPD, and CVD is likely more cost-effective than screening for LC only. The results suggest that the cost-effectiveness of Big-3 screening can be further improved by optimising the target screening population to include individuals who are at risk, especially for CVD. These findings are of great relevance in the ongoing discussion about the cost-effectiveness of LC screening using LDCT. They warrant further research into expanding LC screening to combination screening for the Big-3, focusing on measuring the benefits of COPD and CVD screening using LDCT in future high-quality controlled studies.

## Supplementary Information

Below is the link to the electronic supplementary material.

Supplementary file1 (DOCX 445 KB)

## Abbreviations

COPD: Chronic obstructive pulmonary disease
CVD: Cardiovascular disease
LC: Lung cancer
LDCT: Low-dose CT
QALY: Quality-adjusted life-year
WTP: Willingness-to-pay

## Declarations

### Guarantor

The scientific guarantor of this publication is Maarten IJzerman.

### Statistics and biometry

No complex statistical methods were necessary for this paper.

### Methodology

• prospective

• early HTA

• multicenter study
