# Supplementary material for: Can we increase efficiency of CT lung cancer screening by combining with CVD and COPD screening? Results of an early economic evaluation
Source: Eur Radiol. 2022 Jan 1;32(5):3067–75. doi: 10.1007/s00330-021-08422-7 (PMC9038824; doi:10.1007/s00330-021-08422-7)
Supplement: Supplementary file 1 — Supplementary file1 (DOCX 445 KB) [file 330_2021_8422_MOESM1_ESM.docx]

**Supplementary material**

To conduct the headroom analysis, input data is gathered from literature and public sources. These inputs are adjusted to fit the method of analysis where necessary and finally, the headroom can be calculated for different scenarios.

Supplementary Figure 1, gives an overview of the model framework. All diseases have different stages, with different stage distributions for screening and no-screening.. Following the stage distributions, the health effects in the form of utility and survival (years since diagnosis) and the economic effects (disease cost) are given per disease stage. More details are given for COPD health effects (modelled by smoking cessation) and CVD risk categories and cost calculation. Individuals between ages 50 and 75 are screened in the base-case scenario and the model assumes that all incidences in the Netherlands for lung cancer, COPD and CVD is detected in this target screening population. Supplementary Table 1-Table 4 provides all the evidence used in the model, with a brief description and the source. Where adjustments have been made to the raw input an explanation has been provided in the footnotes.

Supplementary Figure 1 Stage distributions over all stages of three scenarios (A) Current distribution, (B) Realistic screening distribution , including the consequences of each disease stage. LC = lung cancer, CVD = cardiovascular disease, COPD =Chronic Obstructive Pulmonary Disease

*Lung cancer*

Non-small cell (NSCLC) and small cell lung cancer (SCLC) patients (using the incidence below) are divided into TNM stages based on a stage distribution with and without screening. All patients are assigned the average utility, disease cost and survival of the disease stage in which they are detected. Supplementary Table 1 shows they evidence used in the model.

Supplementary Table 1 Evidence for Lung cancer

| **Input** | **Value** | | **Description** | **Source** | |
| --- | --- | --- | --- | --- | --- |
| Incidence | 13,262 | | Annual number of newly diagnosed cases of lung cancer (NSCLC and SCLC) in the Netherlands. | (IKNL, 2017) |  |
| Stage distributions |  | |  |  |  |
| Stage distribution, current diagnosis | Shown in Figure 1, scenario A. | | The proportion of lung cancer patients in each disease stage without any screening, following current diagnostic processes. | [5] |  |
| Expected stage distribution after screening | Shown in Figure 1, scenario B | | The proportion of patients in each stage of lung cancer when screening is implemented. | [5] |  |
| Difference in life expectancy per stage (in years) | **NSCLC**  IA: 1.5  IB: -2.4  IIA: -4.2  IIB: -5.9  IIIA: -7.0  IIIB: -7.7  IV: -8.5  **SCLC**  IA: -7.1  IB: -8.5 | | The probability of survival per lung cancer stage is converted to a difference in life expectancy. ^1^ | [17] |  |
| Utilities | **NSCLC**  IA: 0.72  IB: 0.70  IIA: 0.67  IIB: 0.67  IIIA: 0.65  IIIB: 0.65  IV: 0.62  **SCLC**  IA: 0.72  IB: 0.62 | | The utilities of lung cancer per stage, estimated for a period longer than 12 months. | [18] |  |
| Disease cost per stage | I -II | €17,256 | Stage specific healthcare costs per lung cancer patient.^2^ | [21] |  |
|  | III | €40,561 |  |  |  |
|  | IV | €47,806 |  |  |  |
| ^1^ The 10-year survival probabilities can be transformed into constant yearly mortality rates using Formula 1. Assuming an exponential distribution for the corresponding survival times defined by these rates, the survival times were used to calculate the difference in life expectancy compared to a healthy individual.  Formula 1: Mortality rate = -ln(-survivalprobability+1)/time  Formula 1 was used to calculate the survival rates of non-small cell and small cell lung cancer patients (NSCLC and SCLC), with the probability of survival per stage converted to yearly mortality rates using Formula 1, and then incremental survival, corrected for mean age at diagnosis by subtracting the mean life expectancy of a healthy individual at that age. | | | | |  |
| ^2^ The costs were adjusted from the Australian dollar to Euro with the average exchange rate from 2020 (0.6048 EUR). | | | | |  |

*Chronic Obstructive Pulmonary Disease (COPD)*

COPD patients are divided into GOLD stages. All patients are assigned the average utility, disease cost and survival of the disease stage in which they are detected. The health outcomes of COPD patients who are detected early are assumed to only improve for the individuals who quit smoking. The probability of an individual to have continued smoking abstinence (quit smoking since the quit date in a 2-year follow-up since screening) of 13.9% was used[13]. The proportion of patients who are detected earlier due to screening, but do not stop smoking, do not have any health benefits when they are detected early. Smoking cessation results in slowed progression [8]. To model the health benefit of patients who quit smoking, the number of years in each stage is estimated using the rate of decline of FEV1 per year. For patients who quit smoking (‑31ml/year) and for patients who continue to smoke (-62ml/year)[14] the rate of disease progression was used in combination with the weighted average FEV1 value of Dutch individuals between the ages of 55 and 75 where the average man is 183cm, and the average woman is 169cm tall [15]. Supplementary Table 2 shows the evidence used in the model.

Supplementary Table 2 Evidence for COPD

| **Input** | **Value** | **Description** | **Source** | |
| --- | --- | --- | --- | --- |
| Incidence | 37,300 | Annual number of newly diagnosed cases of COPD in the Netherlands. | [4] |  |
| Stage distributions |  |  |  |  |
| Stage distribution, current diagnosis | Shown in Figure 1, scenario A. | The proportion of COPD patients in each disease stage without any screening, following current diagnostic processes. | [6] |  |
| Expected stage distribution after screening | Shown in Figure 1, scenario B. | The proportion of patients in each disease stage of COPD when screening is implemented. | [8] |  |
| Years per stage used to calculate the difference in health outcomes | | | |  |
| Average number of years to progress from the given stage to the next stage for COPD patients who continue smoking | I: 13.1  II: 12.8  III: 12.5  IV: 7.7 | The patients are assumed to be in the diagnosed stage for the average number of years and thereafter progress to the next stage until the survival years have passed. Unless the survival years are less. Then patients only stay in the diagnosed stage until death. | [4, 14–16] |  |
| Average number of years to progress from the given stage to the next stage for COPD patients who stop smoking | I: 26.1  II: 25.6  III: 25.1  IV: 15.3 | The patients are assumed to be in the diagnosed stage for the average number of years and thereafter progress to the next stage until the survival years have passed. Unless the survival years are less. Then patients only stay in the diagnosed stage until death. | [4, 14–16] |  |
| Utilities per stage | I: 0.897  II: 0.755  III: 0.748  IV: 0.549 | The mean utility scores by COPD severity stage according to GOLD stages. | [20] |  |
| Disease cost per stage | I: €236  II: €293  III: €326  IV: €485 | The annual COPD-related maintenance costs per patient by gender, age and disease severity. ^3^ | [20] |  |
| Survival | I:14.70  II:13.03  III: 9.49  IV:9.49 | The weighted average survival after diagnosis of current and former smokers. ^4^ | [25] |  |
| ^3^ The prevalence fraction according to age and gender was used together with the COPD-related maintenance costs per patient by gender, age and disease severity to calculate an average cost per patient for each disease stage. Only data from patients between 50 and 75 years of age were used, as this is the initial target population group for the headroom analysis. The result is adjusted for 2020 using the Dutch CPI.  ^4^ The life expectancies of 65-year old Caucasian COPD patients are stratified over gender and current and former smokers. The average of these life expectancies is used because the proportion of current and former smokers in the target screening population is not known. In the cited publication, the life expectancies of stage 3 and 4 patients are only presented as an aggregate, therefore the same values will be used for stage 3 and 4 COPD patients. | | | |  |

*Cardiovascular disease (CVD)*

All individuals in the base case target population are assumed to have a basic risk of developing CVD. Therefore, all individuals are categorised into three risk categories based on risk categories in the literature used for inputs in the analysis [1]. The three categories used are I) One or more elevated risk factor, II) One or more major risk factor and III) More than one major risk factor. An elevated risk factor is defined as total cholesterol 5.16 to 6.19 mmol/L (200 to 239 mg/dL), systolic blood pressure 140 to 159 mm Hg, diastolic blood pressure 90 to 99 mm Hg, non-smoker, and non-diabetic. Major risk factors are defined as total cholesterol ≥6.20 mmol/L (≥240 mg/dL), systolic blood pressure ≥160 mm Hg, diastolic blood pressure ≥100 mm Hg, smoker, or diabetic. Smoking is considered to be a major risk factor and it is assumed that being a former smoker is considered to be an elevated risk factor. Therefore the screening population of former and current smokers, used in this study, are all categorised in these three stages. Categories with fewer risk factors were not included. The proportion of CVD patients in each stage is known as well as the risk of experiencing a fatal, non-fatal or no CVD event in each risk category. All patients are assigned the average utility, disease cost and survival of the disease stage in which they are detected with a corresponding event. Supplementary Table 3 provides the evidence used in the model.

The costs of CVD are calculated differently than for lung cancer and COPD. The average cost of individuals at-risk of CVD depends on having fatal, non-fatal or no CVD events and not on the risk category. In this study, myocardial infarction (MI) is the only CVD event considered and the probability of an MI being fatal is the proportion of patients who died of MI over all known MI cases, 40% [26]. In addition to the textual explanation, Supplementary Figure 2 visualises the cost calculations for the three types of events, either with or without screening. There are no costs for individuals without CVD events when no screening strategy is applied, but the once-off costs for fatal CVD events and the once-off and subsequent costs of non-fatal CVD events are incurred. When screening is applied, an annual aspirin treatment cost for patients who do not experience a CVD event is applied for the years since CVD is detected through screening until the individual dies at the same age as an individual without CVD risk factors which corresponds to an age of 89 [1]. Individuals undergoing screening are aged 50 to 75 years and will be screened at different ages and will, therefore, be detected at different ages. The age of CVD detection will on average be 63 (the average of 50 to 75) and is used, within the cost calculation, as the age of detection through screening. For patients experiencing a non-fatal CVD event and those who do not experience a CVD-event, aspirin treatment costs are applied since detection through screening up to the age of MI, which is on average 66.7 years in the Netherlands [27]. Thereafter, the costs incurred for non-fatal MI patients are a once-off cost for patients with a non-fatal MI and annual subsequent costs which are incurred for the number of survival years after MI. Aspirin costs for patients experiencing a fatal CVD event are applied for the years between diagnosis through screening and the average age of fatal MI in the Netherlands given as 82 years [3]. Another cost incurred for patients experiencing a fatal CVD event is a once-off cost for fatal CVD events.


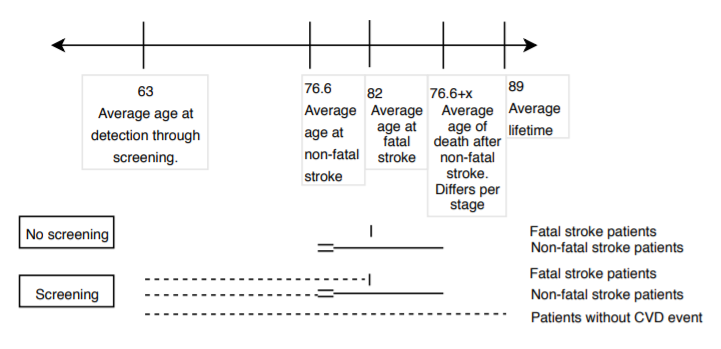


Supplementary Figure 2 Visualisation of CVD costs. CVD = cardiovascular disease

Supplementary Table 3 Evidence for CVD

| **Input** | **Value** | **Description** | **Source** | |
| --- | --- | --- | --- | --- |
| Incidence | 112,300 | Annual number of newly diagnosed cases of CVD in the Netherlands (Angina pectoris, acute myocardial infarction and other chronic ischemic heart diseases). | [3] |  |
| Stage distributions |  |  |  |  |
| Stage distribution, current diagnosis | Shown in Figure 1, scenario A. | The proportion of patients in each CVD disease stage without any screening, following current diagnostic processes. | [1] |  |
| Risk of CVD event per CVD stage, current diagnosis | Shown in Figure 1, scenario A. | The lifetime risk for fatal CVD or major CHD events at age 50, calculated as an average between men and women per stage. | [1] |  |
| Expected stage distribution after screening | Shown in Figure 1, scenario B. | After aspirin treatment, the relative risk reduction for individuals at-risk of CVD to experience a myocardial infarction is 28%. The percentage of patients in each stage who experience a cardiovascular event thus reduces with 28% when screening is implemented. | [7] |  |
| The difference in life expectancy compared to a healthy individual | | | |  |
| Difference in life expectancy of CVD patients per stage (in years) | I: -2  II:-6.5  III: -9.5 | The difference between the median survival in years of patients in each risk group and a patient with all optimal risk factors. | [1] |  |
| Utilities | Patient without a CVD event: 0.83  Patient with a non-fatal CVD event: 0.76 | The utilities of patients with or without non-fatal CVD events.^5^ | [19] |  |
| Disease cost ^6^ |  |  |  |  |
| No CVD event | €146 | Annual cost per person treated with low-dose aspirin. This cost includes drug cost, pharmacist fees and prescription costs.^6^ | [22] |  |
| CVD event costs and remaining costs for one year after non-fatal CVD event | €19,897 | Once-off cost per patient who experienced a non-fatal myocardial infarction. ^6^ | [23] |  |
| Subsequent years after non-fatal CVD event | €1,209 | Cost per patient in the subsequent years after a non-fatal myocardial infarction. ^6^ | [23] |  |
| Fatal CVD event | €1,261 | Costs incurred when a patient experiences a fatal myocardial infarction. ^6^ | [23] |  |
| Survival | I:15.07  II:13.11  III: 11.81 | Average survival years in patients surviving at least 12 months after a non-fatal myocardial infarction. ^7^ | [24] |  |
| ^5^ For CVD, the utilities were obtained from US-based literature [19], where the utility of patients with a non-fatal CVD event is 0.76 and for those without a subsequent non-fatal CV event is 0.83. The probability of a patient from each risk group to experience a cardiac event is reflected in Figure 1 and has been used in combination with these utility values in the analysis. | | | |  |
| ^6^ The costs are adjusted for 2020 using the Dutch CPI. | | | |  |
| ^7^ The survival for former and current smokers are weighted based on the proportion of patients expected to quit smoking after diagnosis. | | | |  |

*Comorbidities*

The last branch of Supplementary Figure 1, “Combinations of diseases based on comorbidities” includes the comorbidities between the Big-3 diseases. The incidence rates or probability of single disease occurrences are assumed to be independent. This analysis makes use of conditional probabilities as a suitable method for including comorbidities, which is the overlap between the two independent diseases.

Supplementary Table 4 Evidence for comorbidities

| Comorbidities in the form of conditional probabilities | | | |
| --- | --- | --- | --- |
| Probability of CVD given COPD | 30% | The probability of having CVD at the time that COPD is diagnosed. | [9] |
| Probability of CVD given lung cancer | 41% | The probability of having CVD at the time that lung cancer is diagnosed. According to literature, the value varies from 36% to 43%, with a weighted average of 41%. ^1^ The probability of having all three diseases is deducted. | [10, 11] |
| Probability of COPD given lung cancer | 52% | The probability of having COPD given lung cancer, from which the probability of having all three diseases is deducted. | [12] |
| Probability of CVD and COPD given lung cancer | 21.32% | The product of the probability of CVD given lung cancer and the probability of COPD given lung cancer. | Assuming conditional independence |
| ^1^ One study of 5 683 lung cancer patients, indicated that 2034 patients had comorbidities including myocardial infarction, congestive heart failure, peripheral vascular disease and Cerebrovascular disease. Another study of 15 900 lung cancer patients, reported a prevalence of 43% among these patients. The weighted average based on the study population has been used. | | | |

*Evidence for scenarios*

Furthermore, Supplementary Table 5 provides the additional input values for the headroom calculated for a smoking population and an aged population. These replace the incidence parameters in Supplementary Table 1-Supplementary *Table* 3.

Supplementary Table 5 Additional input parameters for two screening populations

| **Parameter** | **Description** | **Value** | **Source** |
| --- | --- | --- | --- |
| Incidence of a smoking population | | | |
| Screening population | Number of current smokers between ages 50 and 75 in the Netherlands | 1,265,477 | [28] |
| Incidence of lung cancer | The proportion of smokers who are new cases of lung cancer per year. | 1% ^11^ | [29, 30] |
| Incidence of CVD | The proportion of smokers who are new cases of CVD per year. | 1.9% ^12^ | [3, 31] |
| Incidence of COPD | The proportion of smokers who are new cases of COPD per year. | 1.4% ^13^ | [32] |
| ^11^ The original study cohort with an average age of 50.2, had a lung cancer incidence rate for current smokers of 2.67 per 1000 person-years. The cohort consisted of 3,905 individuals with 32,215 person‑years. The incidence rate was converted to proportion of cases per year. The resulting incidence rate (2.2%) correlates to the prevalence of lung cancer in smokers aged 50-75, therefore this incidence rate is converted to population incidence based on the proportion of LC incidences to prevalence in 2019 in the Netherlands (45.5%).  ^12^ Similarly to the calculation of lung cancer incidence, the prevalence of CVD in occasional and heavy smokers with a mean age of 41.3 from Amiri et al. is given as 11.6%. With Dutch incidence (244 100) and prevalence (1 517 500) rates from 2018 for coronary heart disease, stroke and heart failure, it can be calculated that the annual incidence rates are 16% of the prevalence rate, which is based on the assumption that the ratio of incidence to prevalence remains constant. The product of these proportions (11.6% and 16%) results in 1.9% of Dutch smokers who are new cases of CVD.  ^13^ From a Rotterdam study of individuals aged 45-80 years old, the prevalence of COPD in current smokers is 26%. Combined with Dutch incidence (27 100) and prevalence (496 800) rates, which shows 5.5% incidences of the annual prevalence, gives an incidence of 1.4% of COPD in current smokers. | | | |
| Incidence for a population 60+ years of age | | | |
| Screening population | The number of individuals in the Netherlands over 60 years old. | 25.5% ^14^ | [33] |
| Incidence of lung cancer | The proportion of new cases of lung cancer per year in the population over 60 years old. | 0.2% ^15^ | [33, 34] |
| Incidence of CVD | The proportion of CVD incidence in the Netherlands in a population aged 60 to 90 years. | 4.15% | [35] |
| Incidence of COPD | The proportion of new incidences of COPD in the population over 60. | 0.7% | [36] |
| ^14^ The number of individuals in the Netherlands over 60 years of age is 4 410 843. Which is 25.5% within a population of 17 282 163.  ^15^ The number of new lung cancer cases per 100 000 individuals are given per age group [34]. This is used in combination with the population per age group (using only data from the age groups over 60 years) to determine the absolute number of new cases per year and then expressed as a percentage of individuals over 60 years of age. | | | |

#### General assumptions

Given the purpose of a headroom analysis, assumptions were made and defined such that the benefits of screening are systematically overestimated. Some assumptions are made due to the limited availability of data.

1. 100% sensitivity and specificity for the Big-3 diseases using LDCT.
2. For scenarios A, B and C, all incidences in the Netherlands occur within the target population.
3. A disease is only detected within the first year of getting the disease, thereafter the disease is detected through current diagnostic processes.
4. 100% participation rate amongst invitees.
5. Patients with comorbidities have the QALYs of the most severe disease.
6. All patients with CVD are being treated with low-dose aspirin.
7. CVD patients who undergo screening are, on average, diagnosed at the age of 63, corresponding to an age halfway through the ages in the screening program (50 to 75).
8. Former smokers are assumed to have an elevated risk factor because smoking is considered a major risk factor.

## Supplementary References

1. Lloyd-Jones DM, Leip EP, Larson MG, et al (2006) Prediction of Lifetime Risk for Cardiovascular Disease by Risk Factor Burden at 50 Years of Age. Circulation 113:791–798. https://doi.org/10.1161/CIRCULATIONAHA.105.548206

2. Integraal kankercentrum Nederland (IKNL) (2017) Incidentie Longkanker. https://www.iknl.nl/kankersoorten/longkanker/registratie/incidentie. Accessed 12 Nov 2019

3. Volksgezondheidenzorg.nl (2018) Coronaire hartziekten→Cijfers & Context→Huidige situatie. https://www.volksgezondheidenzorg.info/onderwerp/coronaire-hartziekten/cijfers-context/huidige-situatie#node-prevalentie-en-nieuwe-gevallen-coronaire-hartziekten-huisartsenpraktijk. Accessed 12 Nov 2019

4. Volksgezondheidenzorg.nl (2018) COPD→Cijfers & Context→Huidige situatie. https://www.volksgezondheidenzorg.info/onderwerp/copd/cijfers-context/huidige-situatie#node-prevalentie-en-nieuwe-gevallen-copd-huisartsenpraktijk. Accessed 12 Nov 2019

5. Hassett MJ, Uno H, Cronin AM, et al (2017) Survival after recurrence of stage I–III breast, colorectal, or lung cancer. Cancer Epidemiol 49:186–194. https://doi.org/10.1016/j.canep.2017.07.001

6. Hoogendoorn M, Feenstra TL, Schermer TRJ, et al (2006) Severity distribution of chronic obstructive pulmonary disease (COPD) in Dutch general practice. Respir Med 100:83–86. https://doi.org/https://doi.org/10.1016/j.rmed.2005.04.004

7. Dai Y, Ge J (2012) Clinical Use of Aspirin in Treatment and Prevention of Cardiovascular Disease. Thrombosis 2012:245037. https://doi.org/10.1155/2012/245037

8. Mohamed Hoesein FAA, Zanen P, de Jong PA, et al (2013) Rate of progression of CT-quantified emphysema in male current and ex-smokers: a follow-up study. Respir Res 14:55. https://doi.org/10.1186/1465-9921-14-55

9. Hillas G, Perlikos F, Tsiligianni I, Tzanakis N (2015) Managing comorbidities in COPD. Int J COPD 10:95–109. https://doi.org/10.2147/COPD.S54473

10. Islam KMM, Jiang X, Anggondowati T, et al (2015) Comorbidity and Survival in Lung Cancer Patients. Cancer Epidemiol Biomarkers Prev 24:1079–1085. https://doi.org/10.1158/1055-9965.epi-15-0036

11. Al-Kindi S, Oliveira G (2015) Prevalence of Preexisting Cardiovascular Disease in Patients With Different Types of Cancer: The Unmet Need for Onco-Cardiology. Mayo Clin Proc. https://doi.org/10.1016/j.mayocp.2015.09.009

12. Dutkowska AE, Antczak A (2016) Comorbidities in lung cancer. Pneumonol Alergol Pol 84:186–192. https://doi.org/10.5603/piap.2016.0022

13. van der Aalst CM, van den Bergh KAM, Willemsen MC, et al (2010) Lung cancer screening and smoking abstinence: 2 year follow-up data from the Dutch–Belgian randomised controlled lung cancer screening trial. Thorax 65:600 LP – 605. https://doi.org/10.1136/thx.2009.133751

14. Scanlon PD, Connett JE, Waller LA, et al (2000) Smoking cessation and lung function in mild-to-moderate chronic obstructive pulmonary disease. The Lung Health Study. Am J Respir Crit Care Med 161:381–90. https://doi.org/10.1164/ajrccm.161.2.9901044

15. Hankinson Consulting Inc (2006) SPIROMETRY: Reference Value Calculator. https://www.cdc.gov/niosh/topics/spirometry/refcalculator.html. Accessed 17 Jul 2020

16. Freeman D, Nordyke RJ, Isonaka S, et al (2005) Questions for COPD diagnostic screening in a primary care setting. Respir Med 99:1311–1318. https://doi.org/https://doi.org/10.1016/j.rmed.2005.02.037

17. iknl (2012) Overleving | longcarcinoom; Stadium. http://www.cijfersoverkanker.nl/selecties/dataset_3/img59a2f3e0a1c40

18. Black WC, Gareen IF, Soneji SS, et al (2014) Cost-effectiveness of CT screening in the national lung screening trial. N Engl J Med 371:1793–1802. https://doi.org/10.1056/NEJMoa1312547

19. Lewis EF, Li Y, Pfeffer MA, et al (2014) Impact of cardiovascular events on change in quality of life and utilities in patients after myocardial infarction. A VALIANT Study (Valsartan in acute myocardial infarction). JACC Hear Fail 2:159–165. https://doi.org/10.1016/j.jchf.2013.12.003

20. Hoogendoorn M, Rutten-van Mölken M, Hoogenveen R, et al (2010) Comparing the cost-effectiveness of a wide range of COPD interventions using a stochastic, dynamic, population model for COPD

21. Degeling K, Baxter NN, Emery J, et al (2020) An inverse stage-shift model to estimate the excess mortality and health economic impact of delayed access to cancer services due to the COVID-19 pandemic. medRxiv 2020.05.30.20117630. https://doi.org/10.1101/2020.05.30.20117630

22. Greving JP, Buskens E, Koffijberg H, Algra A (2008) Cost-Effectiveness of Aspirin Treatment in the Primary Prevention of Cardiovascular Disease Events in Subgroups Based on Age, Gender, and Varying Cardiovascular Risk. Circulation 117:2875–2883. https://doi.org/10.1161/circulationaha.107.735340

23. Kievit W, Maurits JSF, Arts EE, et al (2017) Cost-Effectiveness of Cardiovascular Screening in Patients With Rheumatoid Arthritis. Arthritis Care Res (Hoboken) 69:175–182. https://doi.org/10.1002/acr.22929

24. Law MR, Watt HC, Wald NJ (2002) The Underlying Risk of Death After Myocardial Infarction in the Absence of Treatment. Arch Intern Med 162:2405–2410. https://doi.org/10.1001/archinte.162.21.2405

25. Shavelle RM, Paculdo DR, Kush SJ, et al (2009) Life expectancy and years of life lost in chronic obstructive pulmonary disease: Findings from the NHANES III Follow-up Study. Int J COPD 4:137–148. https://doi.org/10.2147/copd.s5237

26. Volksgezondheidenzorg.nl (2019) Beroerte→Cijfers & Context→Oorzaken en gevolgen. https://www.volksgezondheidenzorg.info/onderwerp/beroerte/cijfers-context/oorzaken-en-gevolgen#node-risico-op-sterfte-door-een-beroerte. Accessed 11 Feb 2020

27. Koek HL, Bruin A De, Gast A, et al (2007) Incidence of first acute myocardial infarction in the Netherlands. Neth J Med 65:434–441

28. Centraal Bureau voor Statistiek (2019) Lichte daling aantal rokers onder volwassenen. https://www.cbs.nl/nl-nl/nieuws/2019/12/lichte-daling-aantal-rokers-onder-volwassenen. Accessed 8 Dec 2019

29. Tindle HA, Stevenson Duncan M, Greevy RA, et al (2018) Lifetime Smoking History and Risk of Lung Cancer: Results From the Framingham Heart Study. JNCI J Natl Cancer Inst 110:1201–1207. https://doi.org/10.1093/jnci/djy041

30. Volksgezondheidenzorg.nl (2019) Longkanker→Cijfers & Context→Huidige situatie. https://www.volksgezondheidenzorg.info/onderwerp/longkanker/cijfers-context/huidige-situatie. Accessed 11 Dec 2019

31. Amiri P, Mohammadzadeh-Naziri K, Abbasi B, et al (2019) Smoking habits and incidence of cardiovascular diseases in men and women: findings of a 12 year follow up among an urban Eastern-Mediterranean population. BMC Public Health 19:1042. https://doi.org/10.1186/s12889-019-7390-0

32. Terzikhan N, Verhamme KMC, Hofman A, et al (2016) Prevalence and incidence of COPD in smokers and non-smokers: the Rotterdam Study. Eur J Epidemiol 31:785–792. https://doi.org/10.1007/s10654-016-0132-z

33. van de Pas B (2019) Population of the Netherlands in 2019, by age and gender. In: Statista. https://www.statista.com/statistics/755052/population-of-the-netherlands-by-age-and-gender/. Accessed 8 Dec 2019

34. Kankerregistratie N (2018) Longkanker→Cijfers & Context→Huidige situatie. https://www.volksgezondheidenzorg.info/onderwerp/longkanker/cijfers-context/huidige-situatie. Accessed 8 Dec 2019

35. Leening MJG, Siregar S, Vaartjes I, et al (2014) Heart disease in the Netherlands: a quantitative update. Netherlands Hear J 22:3–10. https://doi.org/10.1007/s12471-013-0504-x

36. Long Alliantie Nederland (2013) Longziekten feiten en cijfers
